# Disentangling the reproductive and metabolic transcriptional responses to diet in *Drosophila melanogaster*

**DOI:** 10.1093/g3journal/jkag020

**Published:** 2026-01-23

**Authors:** M Florencia Camus, Avishikta Chakraborty, Max Reuter

**Affiliations:** Research Department of Genetics, Evolution and Environment, University College London, Gower Street, London WC1E 6BT, United Kingdom; Centre for Life’s Origins and Evolution, University College London, Gower Street, London WC1E 6BT, United Kingdom; Research Department of Genetics, Evolution and Environment, University College London, Gower Street, London WC1E 6BT, United Kingdom; Centre for Life’s Origins and Evolution, University College London, Gower Street, London WC1E 6BT, United Kingdom; Research Department of Genetics, Evolution and Environment, University College London, Gower Street, London WC1E 6BT, United Kingdom; Centre for Life’s Origins and Evolution, University College London, Gower Street, London WC1E 6BT, United Kingdom

**Keywords:** fitness, nutrition, reproduction, nutritional plasticity, life history, gene regulation

## Abstract

Nutrition significantly influences various life-history traits in organisms, impacting decisions about growth, reproduction, and longevity. Accordingly, previous studies in *Drosophila* have demonstrated that diet affects transcription regulation, with many genes exhibiting altered expression, for example, between protein- and carbohydrate-rich diets. It remains challenging, however, to distinguish between metabolic adaptations to the different diets themselves and regulation pertaining to life-history responses to nutrient availability. In this study, we explore the transcriptomic responses of virgin and mated flies to changes in nutritional environments, with the aim to differentiate changes in metabolism from changes due to altered reproductive investment. Using RNA-seq, we show that while males only respond to diet, both nutritional conditions and mating status affect gene expression in females. By comparing responses between males and females and virgin and mated flies, we are able to differentiate between basal dietary responses and reproductive adaptations, with the latter involving 8 times as many genes as the former. We identify GATA family transcription factors and the heat shock factor (*Hsf*) as crucial regulators of diet-dependent reproductive genes. These findings enhance our understanding of the complex interactions between nutrition and reproductive strategies in *Drosophila*.

## Introduction

Nutrition plays a central role in life histories across the animal kingdom, shaping traits such as growth, development, the onset of reproductive maturity, investment in gamete production, and survival. It does so by providing not only building blocks and energetic fuel for organisms, but also environmental cues that are used to make active decisions about how to modulate life histories to maximize expected lifetime fitness. One of the best-studied traits that is heavily dependent on nutrition is reproduction, where the quality and quantity of resources affect investment in energetically costly offspring. In sexual species, females in particular allocate significant resources to the production of a small number of large-sized gametes, and accordingly, nutrition has a large impact on female fecundity (Partridge et al. 1987; Chapman and Partridge 1996). For example, *Drosophila* females increase reproductive output in the presence of a protein source, such as yeast (Simmons and Bradley 1997), with individuals housed in higher protein environments produce up to 4 times as many eggs as females in carbohydrate-rich (and more protein-poor) environments (Lee et al. 2008). Changes in male reproductive investment are typically smaller, but also present (Lee et al. 2008; Camus et al. 2017).

Recent work in fruit flies assessing the transcriptional responses to diet suggests that diet-dependent variation in reproductive output is at least in part due to active regulation of reproductive investment rather than nutrient limitation (Camus et al. 2019). Thus, gene expression changes observed between an optimal protein-rich and a suboptimal carbohydrate-rich diet mirror those observed in response to dietary restriction; similar to limiting dietary input, providing a diet with a suboptimal macronutrient balance triggers an evolutionarily conserved life-history response, where reproduction is actively downregulated. This life-history response relies heavily on the insulin-signaling and especially the TOR pathway (Alic and Partridge 2011; Hoedjes et al. 2017; Camus et al. 2019), via members of the GATA transcription factor family (Dobson et al. 2018; Camus et al. 2019). The presence of these responses makes it difficult to interpret diet-dependent physiological and transcriptional changes, and discriminate which responses are to the diet itself, and which are an indirect consequence of the altered reproductive investment in response to diet.

One way to disentangle these effects on the transcriptome is to combine diet treatments with manipulation of an individual's mating status. In female flies, like in most other organisms, a first mating triggers the transition from a nonreproductive to a reproductive state (Chapman et al. 1995; Sgrò et al. 1998; Liu and Kubli 2003). This also brings about permanent physiological changes to be able to meet reproductive demands (Short and Lazzaro 2010; Flatt 2011; Mirth et al. 2019). For example, studies have documented a drastic shift in nutritional preference before and after mating; while virgin females prefer to feed on carbohydrate-rich foods similar to males, mated females not only consume more food, but switch to preferring diets higher in protein content in order to be able to meet the nutritional demands of egg production (Corrales-Carvajal et al. 2016; Camus et al. 2018). Interestingly, this shift occurs even in genetically sterilized females that do not possess a germline and are unable to produce eggs (Newell et al. 2020). In large parts, these changes are reliant on male-derived sex peptide, which is delivered as part of accessory gland fluids during mating (Chapman et al. 2003; Wigby and Chapman 2005; Ribeiro and Dickson 2010). Several transcriptomic studies have demonstrated that, as expected, the phenotypic changes after mating are associated with widespread responses at the gene expression level between the virgin and mated state (Lawniczak and Begun 2004; Mack et al. 2006; McGraw et al. 2008; Innocenti and Morrow 2009; Parisi et al. 2010; Fowler et al. 2019; Newell et al. 2020; Rodrigues et al. 2021, 2024), but also between low- and high-mating regimes (Gerrard et al. 2013). But again, it is not clear to which degree these changes are a general, hard-wired response to mating and the onset of reproduction or diet-dependent, and if the latter, how expression changes triggered by mating are aligned with responses to diet.

Here, we report experiments that manipulate both diet and mating status in *Drosophila melanogaster* to dissect the complex relationship between nutritional and reproductive responses. Examining gene expression of virgin and mated flies fed either protein- or carbohydrate-rich synthetic diets, we identify and characterize expression changes that represent physiological adaptations to diet (shared by all flies), those that are generic responses to mating (invariant across diets) and those that modulated by both mating status and diet composition. We further compare these classes functionally and in terms of their regulation.

We find several hundred genes respond to mating and nutrition. A large proportion of genes show diet-dependent expression changes that are similar between virgin and mated flies. These genes include a set of core metabolic genes involved in physiological diet responses that are independent of mating status. But we also find evidence for a larger set of genes where expression differences tend to be more pronounced in the mated cohort. Concordant with previous work, we show that the diet response is mainly regulated by GATA transcription factors. However, we also identify *Hsf* (heat shock factor) as an important transcription factor modulating the upregulation of diet-specific reproductive response. Since thermal tolerance depends strongly on nutrient balance and metabolic regulation in *Drosophila*, we incorporated heat-knockdown assays to test whether diet- and mating-induced metabolic shifts were reflected in this key physiological performance trait. In line with a role of *Hsf* in expression regulation, these additional experiments reveal effects of mating status and diet on resistance to heat shock, adding support to an emerging role of heat shock factors in reproduction.

## Materials and methods

### Fly stocks and maintenance

We used *D. melanogaster* from the laboratory population LH_M_. This population has been maintained as a large outbred population for over 400 nonoverlapping generations (Rice 1996; Chippindale et al. 2001). It is kept on a strict 14-d regime, with larval densities kept constant (∼150 to 200 eggs per vial containing ca. 7 mL of media) and a fixed adult population size (56 vials of 16 males and 16 females) at each generation. Flies were reared at 25 °C, under a 12 h:12 h light:dark regime, on a cornmeal–molasses–yeast–agar food medium (200 mL molasses, 24 g agar, 200 g cornmeal, 82 g yeast powder along with 90 mL of nipagen (100 mg/L) and 9 mL of propionic acid for 3 L of media). For all experiments involving capillary feeding, we used high relative humidity (>80%) to minimize evaporation.

### Synthetic diet

For experiments, we used a modified liquid version of the holidic diet described in Piper et al. (2014). This food is prepared entirely from synthetic components to enable precise control over nutritional value (see Supplementary Methods). Two artificial liquid diets were made which differed in the ratio of protein (incorporated as individual amino acids) and carbohydrate (sucrose); with all other nutritional components provided in fixed concentrations. The nutrient ratios (P:C) used were 2:1 and 1:4, with the final concentration of each diet being 32.5 g/L. These ratios were identified in previous work (Camus et al. 2019) as maximizing the reproductive fitness of female and male LH_M_ flies, respectively. We note that P:C ratios may not be directly comparable to standard fly media, as nutrients in synthetic diets appear to be more readily accessible (Piper et al. 2014).

### Phenotypic data collection

#### Diet preference

Flies from each sex were collected as virgins using CO_2_ anesthesia. Triplets of virgins were placed in individual vials containing molasses–yeast–agar culture medium (see Supplementary Methods for full recipe) with no added live yeast. We used triplets of flies as a means to balance between, on the one hand, replication across vials and, on the other hand, between-vial variance and workload (Camus et al. 2017). Twenty vials of triplets were collected for each sex and mating status. All flies were aged for 2 d for them to become sexually mature. Subsequently, females in the “mated” treatment were provided with 3 virgin males (collected at the same time as females) as mating partners. These hextets of flies were left for 5 h to mate, before being re-split by sex and transferred to new vials containing a 0.8% agar–water mixture. Virgin flies were not perturbed during this period and placed on the 0.8% agar medium at the same time as mated flies. The agar–water vials provide water for the flies but have no nutritional value.

In accordance with previous literature using this methodology (Reddiex et al. 2013; Camus et al. 2017), flies were kept in agar–water vials overnight, then supplied with two 5µL microcapillary tubes (ringcaps, Hirschmann) containing amino acid and carbohydrate solutions, respectively. Capillary tubes were replaced daily, and food consumption for each fly trio was recorded for a period of 3 d. As a control, the rate of evaporation for all diet treatments was measured in 6 vials that contained the 2 solution-bearing capillary tubes but no flies, placed alongside the experimental vials in the controlled temperature room. Their average evaporation per day was subtracted from the values of volume consumed in experimental vials to correct diet consumption for evaporation.

#### Nutritional requirement

The setup for nutritional requirement is almost identical to the diet choice experiment, except at the point of synthetic diet allocation. Instead of 2 microcapillary tubes (as per diet choice), flies were supplied with 1 microcapillary tube (ringcaps, Hirschmann) containing 1 of the 2 allocated diets. These diets varied in their protein-to-carbohydrate ratios and captured the following nutritional rails (P:C): 2:1 and 1:4. We had 20 vials with fly triplets per allocated diet and mating status. Capillary tubes were replaced daily, and food consumption for each fly trio was recorded for a total period of 3 d. Flies were exposed to diet treatments in a controlled temperature room (25 °C), 12L:12D light cycle, and high relative humidity >80%. The rate of evaporation for all diet treatments was measured by using 5 vials per diet that contained no flies, placed randomly in the constant temperature chamber. The average evaporation per day was used to correct diet consumption for evaporation.

#### Female reproductive success

We measured the number of eggs produced over a fixed period of time (18 h). This performance proxy is expected to correlate closely with other fitness measures, such as the total number of offspring (Hoffmann and Harshman 1985; Tanaka and Yamazaki 1990). Following the feeding period, trios of virgin or mated females were placed in new agar vials and presented with 3 males of the same age from the LH_M_ stock population. We note that this mating event is the second mating opportunity for the “mated fly” group, and this was done so that we could compare the fecundity of recently inseminated females across both groups. Flies were left to mate/oviposit for 18 h in vials containing ad libitum food corresponding to their diet treatment provided via capillary tubes. All flies were removed after this 18-h mating window. Following removal of the flies, the total number of eggs laid was determined by taking pictures of the agar surface and counting eggs using the software *QuantiFly* (Waithe et al. 2015).

#### Male reproductive success

As a proxy of adult male fitness, we quantified the proportional fertilization success in competitive mating trials. This metric has been previously validated in our laboratory as a reliable indicator of reproductive success, given that total adult progeny production in our population primarily depends on mating success (Pischedda and Rice 2012). Our method closely followed the experimental design outlined in Collet et al. (2016), in which focal experimental males competed against standard competitor males for mating opportunities. After the previously described feeding period, a group consisting of 3 focal males (from both virgin and mated treatments), 3 virgin competitor males, and 6 virgin females was transferred to a fresh vial containing molasses–yeast–agar medium lacking live yeast, the primary food source for both sexes (Sang 1978; Colinet and Renault 2014). Competitor males and females carried an LH_M_ genetic background and were homozygous for the recessive *bw*− eye-color mutation. These competitor flies were raised under identical conditions and matched the age of the focal males. Following a 24-h egg-laying period, adults were removed from the vials. The resulting eggs developed for 12 d, after which adult offspring were counted and classified based on eye color to determine paternity: red-eyed offspring indicated paternity by the focal experimental males, whereas brown-eyed offspring were fathered by the competitor males.

#### Thermal tolerance

Because thermal tolerance is sensitive to metabolic state in *Drosophila*, we measured heat-knockdown time to test whether diet- and mating-dependent metabolic changes translated into a physiological performance trait. Thermal tolerance was assessed in females (Hoffmann et al. 2002). Following the feeding period in triplets, individual females were placed into 4 mL water-tight glass vials, clipped onto a 2-sided rack and immersed in a water bath with temperature set to 39 °C. Thermal tolerance was measured as the time (minutes) it took an individual fly to stop locomotory activity (enter a coma-like state). The experiment was performed over 2 runs, with each run measuring heat knockdown time of 90 individual flies. Thus, a total of 180 flies were measured, corresponding to 15 fly triplets for each combination of diet and mating status.

#### Statistical analysis

Dietary choices were measured as 2 variables, the quantities of protein diet and carbohydrate diet consumed. To assess whether choices differed, we used a multivariate analysis of variance (MANOVA). The main model had protein and carbohydrate consumption as response variables, with mating status, sex, and their interaction as fixed effects. We performed subsequent univariate analysis of variance (ANOVA) to determine which nutrient(s) contributed to the overall multivariate effect. All analyses were performed using the *manova* function.

To examine whether the flies varied in the quantity they consumed of each diet, we used a Gaussian linear model to investigate differences in dietary consumption. We modeled total food consumption as a response variable with diet treatment, sex, and their interaction as fixed effects. We also used Gaussian linear models to analyze the reproductive success of females (total number of eggs produced within an 18-h timeframe following a mating event) and males (proportion of offspring sired by the focal males in a competitive assay), both of which followed a normal distribution. The responses were modeled as a function of mating status, diet composition, and their interaction as categorical fixed effects.

Thermal tolerance data were analyzed with a Gaussian mixed model, fitted with the *lmer* function in the *lme4* package (Bates et al. 2015). The response variable was time (minutes) for flies to become unconscious, with mating status and diet (plus their interactions) as fixed effects. We included the nested term of “side of tank” within “run” of experiment as a random effect.

### Transcriptomic analysis

#### Experimental setup

Flies from each sex were collected as virgins using CO_2_ anesthesia. For the virgin treatment, groups of 6 virgin flies were collected and placed into vials containing culture medium (molasses–yeast–agar) with no added live yeast. For the mated treatment, 3 virgin females and 3 virgin males were placed in individual vials. For both treatments, there was a total of 6 flies in each vial. Flies were left in these vials for a period of 36 h in which the mated treatment had the chance to mate. Following this period, flies were split into triplets and placed on 0.8% agar–water mixture. Agar–water vials provide water for the flies but have no nutritional value. Flies were kept in these vials overnight before being supplied with a microcapillary tube (ringcaps, Hirschmann) containing 1 of the 2 allocated diets.

#### Sample collection and RNA extraction

For each sex, we generated 3 biological replicates for each diet treatment (virgins on protein diet, virgins on carbohydrate diet, mated on protein diet, mated on carbohydrate diet), a total of twelve samples. For each replicate sample, we pooled 4 triplets (a total of 12 flies) to ensure we collected sufficient RNA. Total RNA from whole flies was extracted using the *Qiagen RNeasy Minikit* (Qiagen BV, Venlo, The Netherlands) according to manufacturer's instructions. Quantity and quality of RNA was first inspected using a Nanodrop 2000 spectrophotometer (Wilmington, USA), and later verified using an Agilent Tapestation 2200 (Agilent, USA) at the UCL Genomics facility.

#### Sequencing and read mapping

Library construction and sequencing were performed at the UCL Institute of Child Health Genomics facility. Barcoded cDNA libraries were constructed using the *KAPA Hyper mRNA Library prep kit* (Roche, USA) and mixed at equal concentrations. This multiplexed sample was sequenced (43-bp paired-end reads) on 4 flowcell lanes on an Illumina Nextgen 500 instrument to an average of 18M reads per sample.

Having verified that there was no bias toward particular libraries across the sequencing lanes using the Illumina Basespace online server, we merged reads from all 4 lanes. Adaptors and low-quality base pairs (below quality value of 3) were trimmed using trimmomatic v0.36 (Bolger et al. 2014). Trimmed reads from each sample were independently mapped to the *D. melanogaster* genome release 6.19 using HISAT2 (Kim et al. 2015). Mapped reads were manipulated using *samtools* (Li et al. 2009).

#### Statistical analyses

Exon-level read counts for each annotated gene were obtained using *htseq-count* (Anders et al. 2015), using release 6.19 annotations obtained from the ENSEMBL Biomart, and then summed across all exons within a single gene. Total read counts for each gene for the twelve samples were then used for differential gene expression analysis using the Bioconductor package *edgeR* (Robinson et al. 2010), analyzing each sex separately.

We first removed lowly expressed genes (read count < 2 across all samples). Read count data were then normalized across libraries and expression dispersion parameters calculated using the entire dataset. We tested for differential gene expression between our experimental groups using the negative binomial models implemented in *edgeR*. We fitted a full model where expression of each transcript was a function of mating status, diet and their interaction. The significance of each model term was tested using a specific contrast matrix. To obtain estimates of expression fold changes between the 2 diets for each mating status, we further fitted separate models for each mating status with diet as the sole term.

Gene ontogeny enrichment was performed using the Bioconductor package *clusterProfiler* (Yu et al. 2012). We further compared our list of genes that responded to mating to previous work that has examined transcriptomic responses of changes in mating status. For this, we used the R package *GeneOverlap* (Shen and Sinai 2018) uses contingency table tests to identify greater than expected overlap between gene lists. In order to assess whether genes that showed similar diet responses were regulated by common transcription factors we used the Bioconductor package *RcisTarget* (Aibar et al. 2017), which tests for enrichment of cis-regulatory motifs in 5 kb windows upstream of genes in a given sets. In all analyses, we used a statistical significance threshold of 5% false discovery rate (FDR) (Benjamini and Hochberg 1995 ).

All analyses were performed in R version 3.3.2 (R Core Team 2016).

## Results

### Phenotypic responses

Phenotypic data collected alongside our expression data recapitulated previously described patterns of diet preference and consumption and diet-dependent fitness. Briefly, we observe sexual dimorphism for diet preference, diet consumption and diet-dependent reproductive success, with females both preferring and performing better on high-protein diets and males on high-carbohydrate diets (Supplementary Results 1). Preference assays showed that females, but not males, show a large shift in nutritional preference following mating. While in a choice assay virgin females consumed separately offered protein and carbohydrate diets in relatively small quantities and in proportions that resulted in a protein-to-carbohydrate ratio of 1:2.5 (not dissimilar to the composition chosen by males), mated females consumed a much larger total quantity of food and almost tripled their protein intake, resulting in a significantly altered protein-to-carbohydrate ratio of 1:0.92 (Supplementary Fig. SR1.1A, Supplementary Table SR1.1). In no-choice assays, we found that mating has a significant effect on female total food consumption (Supplementary Fig. SR1.1B, Supplementary Table SR1.2), with mated females consuming more than virgin females. We also found that mated females consumed similar amounts when supplied with high-protein or high-carbohydrate carbohydrate diets, whereas virgin females consumed more on the high-protein diet (Supplementary Fig. SR1.1B, Supplementary Table SR1.2). Measurements of reproductive success showed sex-specific effects, whereby females from the virgin treatment, when provided with males for a mating, were able to lay more eggs than females from the mated treatment, who had already been previously mated and reproductively active, across both diets, but this fitness difference was not diet-dependent. Unlike in females, previous mating did not influence male fitness. Regardless of mating status, however, females performed best on protein and males on carbohydrate (Supplementary Fig. SR1.1, Supplementary Table SR1.3).

### Differential gene expression

After QC, our dataset included expression data for 12,880 genes for males and 9,862 genes for females. For males, we found 139 genes responding significantly in expression to changes in diet; no genes were differentially expressed in relation to mating or its interaction with diet (Table 1). Genes that were differentially expressed in relation to diet included a number of that are associated with male ejaculates, specifically accessory gland proteins (24A4, 53C14a, 53C14c, 53Ea, 62F, 63F, 76A), seminal fluid proteins (24Bb, 33A3, 53C14a, 60F, 87B), and serpins (28F, 38F, 77Bb), as well as *Seminal metalloprotease-1*, *Ejaculatory bulb protein*, and *Ejaculatory bulb protein II* (see online archived dataset 6 for a full list of genes). All of these are downregulated on protein-rich compared to carbohydrate-rich food, in line with the diet-specific reproductive investment observed in our phenotypic assays.

**Table 1.** Numbers of genes that do or do not show significant differential expression in response to Mating, Diet, and their interaction in males and females.

significance (FDR < 0.05)					
Mating	Diet	Mating × Diet	n. genes male	n. genes female	Category
–	–	–	9,723	8,263	
Y	–	–	0	345	Mating
–	Y	–	139	651	Diet
Y	Y	–	0	110	Diet + Mating
–	–	Y	0	3	Diet × Mating
–	Y	Y	0	9	Diet × Mating
Y	Y	Y	0	2	Diet × Mating

Based on the significance of the 3 predictor terms, genes were classified into 4 functional categories (last column).

Transcriptomic responses in females were considerably more complex (Table 1, and see online archived dataset 5 for a full list of genes). We identified 651 genes that showed differential expression between the diet treatments (“Diet” set), 345 genes that showed differential expression between virgin and mated flies (“Mating” set), 110 genes that showed additive expression changes in response to both diet and mating (“Diet + Mating” set) and 14 genes that showed a significant diet-by-mating interaction (“Diet × Mating” set), some in combination with additive diet and/or mating effects.

**Fig. 1.**
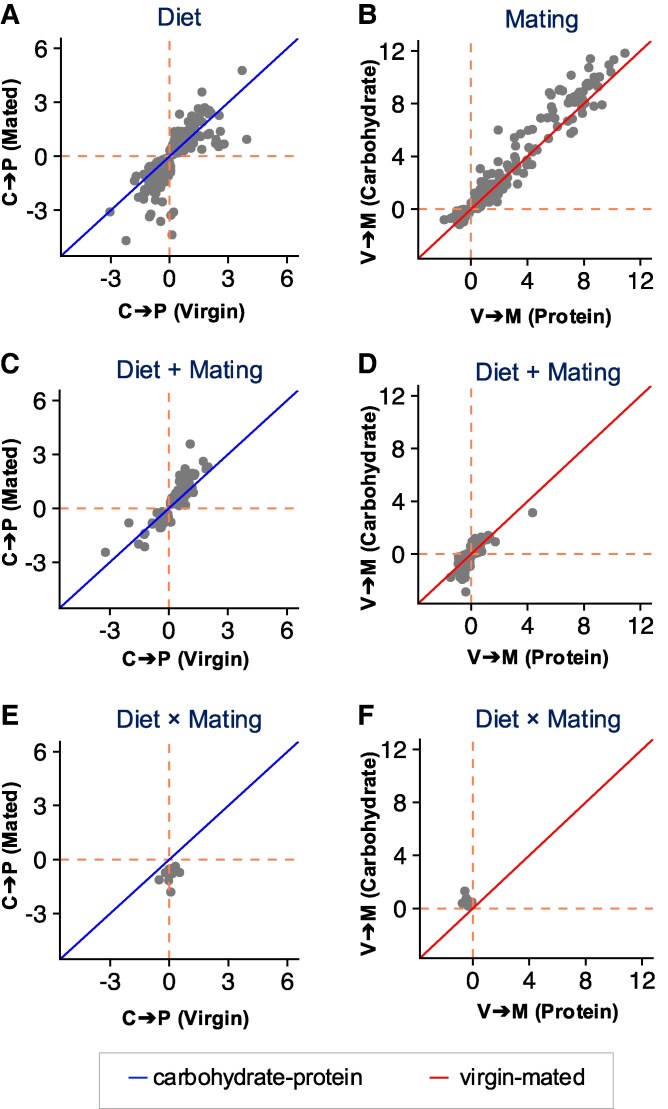
Correlations in log_2_-fold changes across treatments for genes classified as showing: only a diet effect (Diet) or only a mating effect (Mating) (first row of panels), an additive effect of both (Diet + Mating, second row) or a diet-by-mating interaction (Diet × Mating, third row). Fold changes in the left column are from the carbohydrate-rich to protein-rich diet and shown between virgins (*x* axis) and mated females (*y* axis), fold changes in the right column are from virgins to mated females and shown between the protein-rich diet (*x* axis) and the carbohydrate-rich diet (*y* axis). Orange dashed lines indicate zero-fold change for the 2 dimensions, blue and red diagonal lines designate equal fold change in the 2 conditions (slope = 1).

**Fig. 2.**
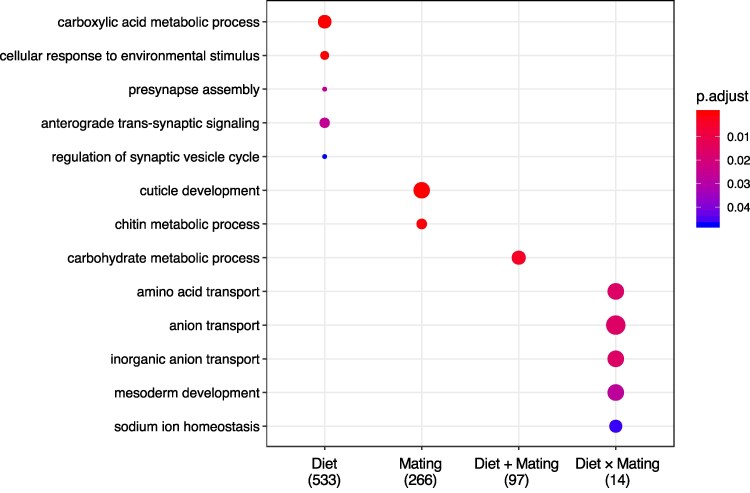
GO enrichment for the transcriptomic response to changes in diet and mating status. Enrichment analyses for “biological process” were performed for all gene categories indicated on the *x* axis, and *P*-values were adjusted for FDR < 0.05 (“p.adjust”).

Genes in the Diet set represent a core metabolic response to nutritional composition. Expression changes occur in both directions (up- and downregulation when going from a carbohydrate to protein environment, Fig. 1a) and carbohydrate-to-protein fold changes are positively correlated between virgin and mated flies (*r* = 0.826, *P* < 0.001). GO analysis showed that Diet genes are significantly enriched for terms that relate to neuronal signaling and response to stimulus (Fig. 2).

In line with female Diet genes being part of a basal metabolic response to diet, genes in the set significantly overlap with the genes that we identify here as showing a significant diet effect across virgin and mated males (overlap = 41 genes, *P* < 0.001). Furthermore, we detect significant overlap with the gene sets identified previously (Camus et al. 2019), when analyzing gene expression only across the mated females and males of this dataset. Most importantly, genes of the Diet set identified here overlap with genes that we previously found to have concordant diet responses between mated males and females (D category in Camus et al. 2019, overlap = 305 genes, *P* < 0.001, Table 2). This indicates that many of the genes that show similar diet responses between virgin and mated females also show similar responses between mated females and males. But in addition, the Diet set identified here also includes genes previously shown to have female-specific responses to diet among mated individuals (D × S category in Camus et al. 2019: overlap = 15 genes, *P* < 0.001) as well as those that respond differently to diet between mated females and males (D + D × S in Camus et al. 2019: overlap = 70 genes, *P* < 0.001, Table 2).

**Table 2.** Gene overlap analysis between expression categories for female-expressed genes obtained from our current analysis (see Table 1) and categories defined in previous work (Camus et al. 2019) investigating expression responses to diet across both sexes (“D,” “D × S,” “D + D × S”) and in genes with female-limited expression (“fem. lim.”).

		Current female-specific dataset			
		Diet	Mating	Diet + Mating	Diet × Mating
		651	345	110	14
Camus et al. (2019)	D 639	**305**	19	**47**	**7**
	D × S 51	**15**	5	**5**	0
	D + D × S 116	**70**	2	**31**	**5**
	fem. lim. 165	8	**45**	0	0

Statistically significant excess overlap is indicated in bold face.

Genes in the Mating set represent female responses to fertilization. Fold-changes from virgin to mated state are mostly positive (indicating increased expression after mating) and—as expected—are highly correlated across diets (Fig. 1b). Gene Ontology (GO) enrichment analysis revealed that mating-dependent genes enriched for terms related to cuticle development, chitin catabolic process and cell adhesion (Fig. 2).

The set of mating-dependent genes identified here overlapped significantly with those previously reported as showing differential expression between mated and virgin females in the abdomen (Fowler et al. 2019; overlap = 19 genes, *P* < 0.001), but not in the head-thorax (overlap = 102 genes, *P* = 0.5). They are also significantly enriched for previously identified reproductive genes with female-limited expression (Camus et al. 2019; overlap = 45 genes, *P* < 0.001, Table 2).

Genes in the small Diet + Mating category show concordant responses to mating between diets (*r* = 0.884, *P* < 0.001, Fig. 1c) and concordant responses to diets between mating treatments (*r* = 0.811, *P* < 0.001, Fig. 1d, possibly with some undetected carbohydrate-specific expression changes following mating). Functionally, Mating genes were significantly enriched for carbohydrate metabolic processes (Fig. 2). For this set, we further find significant overlap with genes previously shown to have similar diet responses across the sexes (D category in Camus et al. 2019: overlap = 47 genes, *P* < 0.001, Table 2), as well as sex-specific diet-dependent regulation (D × S category in Camus et al. 2019: overlap = 5 genes, *P* < 0.001; D + (D × S): overlap = 31 genes, *P* < 0.001, Table 2).

Finally, for the Diet × Mating interaction category, we find patterns of fold change that indicate genes that are upregulated in the carbohydrate environment in mated flies only, while being unaffected by diet in virgins (Fig. 1e and 1f). Functionally, Diet × Mating genes are significantly enriched for biological processes of amino acid transport (Fig. 2). Despite its small size, we found significant overlap between the gene set and those previously shown to have concordant diet responses across the sexes (D category in Camus et al. 2019: overlap = 7 genes, *P* < 0.001, Table 2) and those showing opposing diet-dependent regulation between males and females (D × S category Camus et al. 2019: overlap = 5 genes, *P* < 0.001, Table 2).

### Expression regulation

We used analyses that detect the enrichment of binding motifs in the regulatory regions of sets of genes to infer the transcription factors that are the best candidates for driving the observed patterns of differential expression (Table 3, see online archived datasets 6 and 7 for full results).

**Table 3.** Enriched transcription factor binding motifs for each of the 4 expression categories defined in Table 1.

	Category	Transcription factor prediction
Male	Diet	GATA family (GATAd, GATAe, grn, pnr, srp)
Female	Diet	GATA family (GATAd, GATAe, grn, pnr, srp)
	Mating	Hsf, Stat92E, Syb, ftz-f1, Blimp-1,
	Diet + mating	GATA family (GATAd, GATAe, grn, pnr, srp)
	Diet × mating	Hnf4, Est96B, Hr4, CG7786, bigmax

For males, the genes responding in expression to diet are enriched for transcription factors of the GATA family, such as *grain* (*grn*), *GATAd*, *GATAe*, *pointer* (*pnr*), and *serpent* (*srp*). In the more complex female dataset, we found both the Diet and the Diet + Mating sets to be enriched for regulation by transcription factors of the GATA family, similar to regulation in males. Genes in the Mating set were enriched in binding motifs for *Heat shock factor* (*Hsf*), as well as *Signal-transducer and activator of transcription protein at 92E* (*Stat92E*), *Synaptobrevin* (*Syb*), *ftz transcription factor 1* (*ftz-f1*), and *Blimp-1*. Finally, genes in the Diet × Mating category had upstream regions enriched for binding motifs for *Hepatocyte nuclear factor 4* (*Hnf4*), *Ets96B*, *Hormone receptor 4* (*Hr4*), CG7786, and *bigmax*.

### Thermal tolerance

**Fig. 3.**
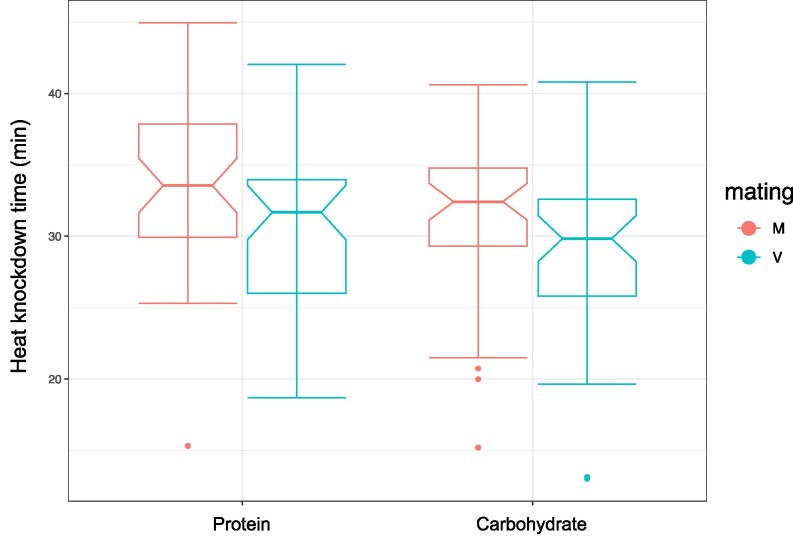
Boxplots of heat knockdown response (time to knock down, *y* axis) of mated (red) and virgin (blue) females fed a protein-rich or a carbohydrate-rich diet (*x* axis). Bold lines indicate medians, boxes the interquartile range, and whiskers the most extreme values within 1.5 interquartile ranges. Dots indicate data points beyond this range.

With gene expression in response to mating enriched for regulation by *Hsf*, we assessed thermal tolerance in virgin and mated females on the 2 diets. The data showed that overall, mated females were significantly more heat-tolerant than virgin females (Supplementary Table SR1.4, Fig. 3). Additionally, we found that females fed on high-protein diet were also significantly more heat-tolerant than flies fed on high-carbohydrate diet (Supplementary Table SR1.4, Fig. 3). Both effects were of similar size (coefficients ± SE: mating = −2.86 ± 1.19, diet = −2.16 ± 1.17) and additive (mating status-by-diet interaction not significant, Supplementary Table SR1.4). Accordingly, out of the 4 treatment groups, mated females fed with the protein-rich diet had the highest heat tolerance levels, with mated females on the carbohydrate-rich diet having a similar thermal tolerance to virgin flies on protein-rich diet and virgins fed the protein-rich diet having the lowest heat tolerance (Fig. 3).

## Discussion

In this study, we aimed to clarify the relationship between nutrition and reproduction in *Drosophila*. We placed virgin and mated flies into 2 nutritional environments that differed in their protein-to-carbohydrate ratios and had previously been shown to represent the female- and male-optimal diets (Camus et al. 2019). We performed phenotypic measurements to confirm that our diet manipulations replicated previously described patterns and then investigated the transcriptome across these treatments to separate genes involved in nutritional responses from those that are regulated in line with reproductive investment.

Our phenotypic experiments replicated previous findings, thus validating our approach. In no-choice assays, we found the expected patterns of sex-specific reproductive success on the 2 types of diet across virgin and mated flies (Sgrò et al. 1998; Chapman et al. 2003; Alic and Partridge 2011), where females are more fecund on protein-rich than carbohydrate-rich food while males gain greater fertilization success on the carbohydrate-rich food. Choice assays provided evidence for a limited response to mating of male diet preference (Piper et al. 2014; Camus et al. 2017, 2018), compared to a large shift of diet preference in females (Ribeiro and Dickson 2010; Camus et al. 2018).

Transcriptome analysis revealed sex differences in diet- and mating-dependent gene expression that match those observed in feeding behavior. Males show a very restricted change in expression between the 2 types of diet, but this response is independent of mating status, and mating in itself does not result in any transcriptomic changes. Females, in contrast, not only share the core dietary response that is seen in males but also exhibit profound changes in gene expression patterns that are triggered by mating, as well as some mating-dependent changes in their responses to diet.

The fact that the male diet response includes many ejaculate-associated genes whose expression is regulated in parallel to reproductive output suggests that male reproductive investment is modulated in line with the nutritional environment. This contrasts with the absence of any plasticity in response to mating status, implying that adult males are in a reproductively active state whether mating takes place or not. This fact is consistent with an onset of spermatogenesis already in the pupal state (Gärtner et al. 2014). It should be noted, however, that the absence of a transcriptional mating response in males reported here is at least somewhat at odds with the only 2 previous studies that have compared gene expression in virgin and mated males. Ellis and Carney (2010) measured gene expression in heads using microarrays and found a modest response, with 47 genes differentially expressed between males that had mated vs controls that had not been presented with a female. Fowler et al. (2019) used RNA-seq to assay gene expression in 2 tissues, head-thorax and abdomen. While they detected no expression change in head-thorax, they found a much larger response to mating in the abdomen with 2,068 differentially expressed genes (Fowler et al. 2019). The fact that these effects of mating were not detected in our study could be due to signals being obscured in the whole flies (rather than specific tissues) analyzed here. Because whole flies contain many tissues with distinct transcriptional profiles, tissue-specific mating responses may be diluted or masked in our dataset. Specifically, signals arising from small or specialized tissues could therefore go undetected, even if biologically meaningful. Our results should thus be viewed as organism-level patterns, and future tissue-resolved analyses will be needed to pinpoint where mating responses occur. Furthermore, differences between our and previous results could arise due to the timing of sample collection. The 2 other studies were conducted by taking samples soon after an observed mating (2 h for Ellis and Carney, 3 h for Fowler et al.). It is possible that expression changes induced by mating are transient and could then have been missed in our design, where males were sampled from mating groups independently of the occurrence of recent mating events. It is worth noting, however, that the differential expression of accessory and seminal fluid proteins between mated and virgin males that was detected by Fowler et al. (2019), presumably due to replenishment after mating, is also apparent in our dataset, but as a correlate to differences in overall reproductive investment between the diet treatments.

In contrast to results in males, the large transcriptional response to mating that we observe in females is in line with previous results. For example, Fowler et al. (2019) found 125 genes to be differentially expressed in response to mating in the female abdomen and 2,040 genes in the head-thorax. Similarly, Delbare et al. (2017) compared the mating response across lines of different geographic origins and found a shared set of 272 genes that were consistently differentially expressed between mated and virgin females (in addition to a further 77 genes that showed differential expression in specific combinations of female and male origin). Beyond these quantitative similarities, we also find correspondence between the specific genes that we and previous work identify as being regulated in response to mating. Thus, genes in our Mating category overlap significantly with those that Fowler et al. (2019) detected as differentially expressed between the abdomens of mated and virgin females. We note that a similar result was not obtained when comparing our gene set with Fowler et al.'s differentially expressed genes from the head-thorax. However, it is worth noting that their gene set is very large (more than 2,000 genes) and contains a smaller proportion of genes that are also detected as mating-responsive in other studies than the thorax set (29% vs 65% of genes also detected elsewhere, Fowler et al. 2019).

While expression changes associated with mating have been well studied, we were also able to assess the effect of diet alongside and in combination with the mating response. Doing so, we could generate several new insights. First, our analysis allowed us to extend the inference of a core metabolic response to macronutrient composition from mated females and males (Camus et al. 2019) to virgin females and males. The existence of such a fundamental physiological response to diet is not surprising, but the fact that it also occurs in individuals that haven’t yet engaged in reproduction corroborates this finding. More interestingly, our analyses revealed that genes that are part of the core response to diet composition, shared between virgin and mated females as well as males, are significantly enriched for binding sites for transcription factors of the GATA family. This could seem surprising, as members of this transcription factor family have been associated with a number of reproductive and aging phenotypes. Thus, GATA transcription factors are involved in the regulation of reproductive genes in fruit flies (Lossky and Wensink 1995) and mosquitoes (Attardo et al. 2003), as well as in the aging response to dietary restriction in *Caenorhabditis elegans* (Budovskaya et al. 2008) and regulation associated with dietary restriction and rapamycin treatment in flies (Alic and Partridge 2011). While these functions should not apply to virgins, our results are more in line with previous studies that implicated GATA factors in the regulation of physiology and feeding, especially in response to sugar (Erickson et al. 2020). Further work will be needed to investigate whether the regulation of life-history decisions and more general physiology are distinct functions of GATA factors, or whether regulators of physiology are deployed in different contexts.

Beyond the core response to diet composition, the analyses presented here suggest that diet composition and mating responses act largely independently and additively, with the number of genes for which we detect a significant diet-by-mating status interaction being very small. This is not what we had expected, as we had predicted that diet and mating would act synergistically. Specifically, we had expected that a significant number of genes would show differential responses to diet in virgin and mated females, with a larger increase in expression under the more favorable conditions in mated females than in virgins, matching feeding rates and reproductive investment. The fact that we observe an additive effect of mating would imply that the mating response is largely qualitative (mating induces the production of gene products) rather than quantitative (the amount of gene product produced scales with reproductive output).

When interpreting the above results, however, it is important to keep in mind that the number of genes with a significant interaction effect between diet and mating status is likely to be limited by the statistical power to pick up these more subtle effects. It is interesting in this respect to contrast the findings of our factorial analysis with results obtained by separate univariate analyses performed in each mating regime (see Supplementary Results 2). These analyses detect a large excess of differentially expressed genes in the mated compared to the virgin mating regime (887 vs 102 genes). Of course, this result is also limited by statistical power, which here reduces the ability to detect shared diet-specific regulation when analyzing 2 datasets of half the size compared to the joint analysis in the main text. So taken together, these results suggest that there is a sizeable set of genes that show quantitative differences between mated and virgin flies in their level of diet-specific regulation, where fold changes are larger in the former and smaller in the latter. These differences in differential expression are small enough to not result in significant interaction effects in the joint analysis, but large enough to lead to contrasting significance in the separate analyses.

It is interesting in this context that a previous study investigating the interplay between diet and reproduction did find a relatively large number of genes with a significant interaction between the 2 treatments. Rodrigues et al. measured gene expression in *D. melanogaster* females that either reproduced normally or were prevented from reproducing (but not mating) by having their germline genetically ablated (Rodrigues et al. 2024). These were fed with diets that differed in the level of yeast extract, as a way to manipulate protein content (Rodrigues et al. 2024). As previous studies comparing flies with and without germline (Parisi et al. 2010; Rodrigues et al. 2021), they found large numbers that were expressed differently between the 2 reproductive states as well as those that responded to the diet treatments. Unlike here, however, they also found hundreds of genes that responded differently to diet in fertile and sterile females. Most importantly, germline-less females would increasingly activate genes involved in lipid turnover as protein levels in the food increased. These results are not immediately relevant in the context of the questions we are asking here, as both the reproductive states (virgin vs mated here, sterile and mated vs reproductively active in Rodrigues et al. 2024) and the diet treatments (isocaloric synthetic foods with different composition here, increasing addition of yeast-based protein in Rodrigues et al. 2024) are very different. Yet, Rodrigues et al.'s result demonstrates that reproductive state and diet jointly shape the female transcriptome, corroborating the view that interactive effects were potentially missed here.

Another interesting result from the present work is the identification of *Heat shock factor* (*Hsf*) as enriched in the regulation of genes related to reproduction and diet. As their name suggests, heat shock factors were first described as drivers for responses to thermal stress (Ritossa 1962). However, there have been several studies suggesting roles in important biological functions in the absence of stress. Most notably, *Hsf* is required for oogenesis in *Drosophila* in a way that does not depend on the expression of heat shock proteins (Jedlicka et al. 1997) and shows its highest level of tissue-specific expression in ovaries (Leader et al. 2018). A similar role for heat shock factors in fecundity has been described in mice (Xiao et al. 1999; Christians et al. 2000; Akerfelt et al. 2010), where they also have been shown to play a role in sperm production (Widlak and Vydra 2017). In line with the role of heat shock factors in reproduction, a large number of *Hsf* binding sites in *Drosophila* are associated with genes that are not involved in the heat shock response, and *Hsf*-regulated genes are enriched for GO categories associated with gamete formation and oogenesis (Gonsalves et al. 2011). Considering these previous findings, it seems that *Hsf* is involved in the diet-dependent regulation of female reproduction, binding to genes that are differentially regulated in response to the macronutrient composition of the food consumed by females.

Not much is known about the mechanistic links between *Hsf* and diet. Work in rodent models has also shown that the amino acid glutamine is involved in the activation of *Hsf* (Brasse-Lagnel et al. 2009), while experiments with the nematode *C. elegans* have demonstrated links between *Hsf* and the insulin-signaling network, with inputs from both being required for development, stress response, survival, and reproduction (Barna et al. 2012). In *Drosophila*, larvae raised in high-protein environments are better able to tolerate thermal stress than flies raised in carbohydrate-rich environments (Andersen et al. 2010; Sisodia and Singh 2012). A recent study further found that males fed a control food take longer to be knocked down by heat than males fed a restricted diet rich in complex carbohydrate–cellulose (Bak et al. 2025). However, no study to date has examined how mating status interacts with the dietary environment to shape the heat shock response. Our experiments confirmed a role of diet in modulating heat resistance, showing that flies fed a protein-rich diet were more heat tolerant than flies fed a carbohydrate-rich food, possibly because a protein-rich diet favors the production of protective proteins such as heat shock proteins, antioxidants, and enzymes involved in repair. Moreover, we found that independently of the diet treatment, mated flies exhibited better tolerance to heat compared to virgins, suggesting that the metabolic conditioning induced post-mating extends beyond egg or offspring production, generating additional protective mechanisms without the trade-offs previously assumed. Future studies should dissect this further by editing 1 or more known heat shock factors to determine which are specifically involved in oogenesis vs those contributing to stress tolerance. It is unclear whether and to which degree these 2 roles are related. Previous work has proposed that the role of *Hsf* in oogenesis is independent of the heat shock response (Jedlicka et al. 1997), yet our observations suggest some possible links.

In conclusion, we were able to delineate a core transcriptional response to diet, present in both males and females, from a female-specific transcriptomic program in response to mating that potentially also shows diet-dependent components. The transcriptional patterns in response to mating are consistent with metabolic shifts that support oogenesis, even though this connection will require experimental validation. By dissecting the nutritional response into basal and reproduction-related components, we also identify several transcription factors including members of the GATA family and *Hsf* as potential regulators. Our work provides further understanding of how organisms are able to adapt their physiology to cope with changes in the internal and external state.

## Supplementary Material

jkag020_Supplementary_Data

## Data Availability

RNA-seq raw data associated with this study are available on the Short Read Archive under accession PRJNA1346554. Files with the data for phenotypic assays (VM-datafile1-fitness.csv, VM-datafile2-preference.csv, VM-datafile3-heat.csv), lists of differentially expressed genes (VM-datafile4-genelist-female.xlsx, VM-datafile5-genelist-male.xlsx), and enriched transcription factor binding sites (VM-datafile6-genelist-female-TF.xlsx, VM-datafile7-genelist-male-TF.xlsx) are available on the GSA figshare: https://doi.org/10.25387/g3.30375490. Media recipes can be found in the Supplementary Methods, while Supplementary Results 1 provides figures and tables relating to the phenotypic assays associated with our study, and Supplementary Results 2 contains text, figures, and tables relating to an alternative analysis of the transcriptomic data, based on separate models applied to data from virgin and mated females. Supplemental material available at G3 online.
