# Supplementary material for: Disentangling the reproductive and metabolic transcriptional responses to diet in Drosophila melanogaster
Source: G3 (Bethesda). 2026 Jan 23;16(4):jkag020. doi: 10.1093/g3journal/jkag020 (PMC13042296; doi:10.1093/g3journal/jkag020)
Supplement: jkag020_Supplementary_Data [file jkag020_supplementary_data.zip › Supplementary_Methods_G3-2025-406300.docx]

**Disentangling the reproductive and metabolic transcriptional responses to diet in *Drosophila melanogaster***

M. Florencia Camus, Avishikta Chakraborty and Max Reuter

**Supplementary Methods: Composition of diets**

**Table SM.1:** Composition of the standard cornmeal-agar-molasses diet. The recipe is given for 3l of media.

| Ingredient | Quantity |
| --- | --- |
| Molasses | 200ml |
| Agar | 24g |
| Cornmeal | 200g |
| Yeast powder | 82g |
| Nipagin (100mg/L) | 90ml |
| Propionic Acid | 9ml |

**Table SM.2:** Essential and non-essential amino acid stock solutions.

| Amino acid stock solution |  | **(g/200 ml)** | |
| --- | --- | --- | --- |
| **Essential amino acid** | | | |
| F (L-phenylalanine) |  | **3.03** |  |
| H (L-histidine |  | **2.24** |  |
| K (L-lysine) |  | **5.74** |  |
| M (L-methionine) |  | **1.12** |  |
| R (L-arginine) |  | **4.70** |  |
| T (L-threonine) |  | **4.28** |  |
| V (L-valine) |  | **4.42** |  |
| W (L-tryptophan) |  | **1.45** |  |
| **Non-essential amino acid** | | | |
| A (L-alanine) |  | **5.25** |  |
| D (L-aspartate) |  | **2.78** |  |
| G (glycine) |  | **3.58** |  |
| N (L-asparagine) |  | **2.78** |  |
| P (L-proline) |  | **1.86** |  |
| Q (L-glutamine) |  | **6.02** |  |
| S (L-serine) |  | **2.51** |  |
|  |  |  |  |
|  |  |  |  |

| **Table SM.3:** Recipe for 200ml of protein solution   \|  \|  \|  \| **Total volume 200ml** \| \| --- \| --- \| --- \| --- \| \|  \| L-ile \| Powder \| 348mg \| \|  \| L-leu \| Powder \| 492mg \| \|  \| L-tyr \| Powder \| 252mg \| \|  \|  \|  \|  \| \|  \| cholesterol \| 20mg/ml in EtOH \| 3ml \| \|  \|  \|  \|  \| \|  \| CaCl2 \| 1000x \| 200ul \| \|  \| MgSO4 \| 1000x \| 200ul \| \|  \| CuSO4 \| 1000x \| 200ul \| \|  \| FeSO4 \| 1000x \| 200ul \| \|  \| MnCl2 \| 1000x \| 200ul \| \|  \| ZnSO4 \| 1000x \| 200ul \| \|  \| H_2_O \|  \| Up to 50ml \| \|  \| Total volume before autoclaving \| \| 50 ml \| \|  \|  \|  \|  \| \|  \| buffer \| 10x acetate buffer base \| 20ml \| \|  \|  \|  \|  \| \|  \| nucl/lipid soln \| 125x stock \| 1.6ml \| \|  \|  \|  \|  \| \|  \| Yaa solutions \| essential amino acid stock solution (EAA) \| 18.154ml \| \|  \|  \| non essential amino acid stock solution (NEAA) \| 18.154ml \| \|  \|  \| Na glutamate solution (100mg/ml) \| 5.464ml \| \|  \|  \| Cys solution (50mg/ml) \| 1.584ml \| \|  \|  \|  \|  \| \|  \| Vitamin stock \| 47.6x stock \| 4.2ml \| \|  \|  \|  \|  \| \|  \| folic acid stock \| 1000x stock \| 200ul \| \|  \|  \|  \|  \| \|  \| Propionic acid \|  \| 1.2ml \| \|  \|  \|  \|  \| \|  \| Nipagin \| 100 g/l stock in 95% EtOH \| 3ml \| \|  \|  \| Make to total volume of 200ml with H_2_O \|  \|   **Table SM.4:** Recipe for 200ml of carbohydrate solution   \|  \|  \|  \| **Total volume 200ml** \| \| --- \| --- \| --- \| --- \| \|  \| sucrose \| To match protein 1:1 \| 6.5g \| \|  \|  \|  \|  \| \|  \| cholesterol \| 20mg/ml in EtOH \| 3ml \| \|  \|  \|  \|  \| \|  \| CaCl2 \| 1000x \| 200ul \| \|  \| MgSO4 \| 1000x \| 200ul \| \|  \| CuSO4 \| 1000x \| 200ul \| \|  \| FeSO4 \| 1000x \| 200ul \| \|  \| MnCl2 \| 1000x \| 200ul \| \|  \| ZnSO4 \| 1000x \| 200ul \| \|  \| H_2_O \|  \| Up to 50ml \| \|  \| Total volume before autoclaving \| \| 50ml \| \|  \|  \|  \|  \| \|  \| buffer \| 10x acetate buffer base \| 20ml \| \|  \|  \|  \|  \| \|  \| nucl/lipid soln \| 125x stock \| 1.6ml \| \|  \|  \|  \|  \| \|  \| Vitamin stock \| 47.6x stock \| 4.2ml \| \|  \|  \|  \|  \| \|  \| folic acid stock \| 1000x stock \| 200ul \| \|  \|  \|  \|  \| \|  \| Propionic acid \|  \| 1.2ml \| \|  \|  \|  \|  \| \|  \| Nipagin \| 100 g/l stock in 95% EtOH \| 3ml \| \|  \|  \| Make to total volume of 200ml with H_2_O \|  \| |  |  |  |
| --- | --- | --- | --- | --- | --- | --- | --- | --- | --- | --- | --- | --- | --- | --- | --- | --- | --- | --- | --- | --- | --- | --- | --- | --- | --- | --- | --- | --- | --- | --- | --- | --- | --- | --- | --- | --- | --- | --- | --- | --- | --- | --- | --- | --- | --- | --- | --- | --- | --- | --- | --- | --- | --- | --- | --- | --- | --- | --- | --- | --- | --- | --- | --- | --- | --- | --- | --- | --- | --- | --- | --- | --- | --- | --- | --- | --- | --- | --- | --- | --- | --- | --- | --- | --- | --- | --- | --- | --- | --- | --- | --- | --- | --- | --- | --- | --- | --- | --- | --- | --- | --- | --- | --- | --- | --- | --- | --- | --- | --- | --- | --- | --- | --- | --- | --- | --- | --- | --- | --- | --- | --- | --- | --- | --- | --- | --- | --- | --- | --- | --- | --- | --- | --- | --- | --- | --- | --- | --- | --- | --- | --- | --- | --- | --- | --- | --- | --- | --- | --- | --- | --- | --- | --- | --- | --- | --- | --- | --- | --- | --- | --- | --- | --- | --- | --- | --- | --- | --- | --- | --- | --- | --- | --- | --- | --- | --- | --- | --- | --- | --- | --- | --- | --- | --- | --- | --- | --- | --- | --- | --- | --- | --- | --- | --- | --- | --- | --- | --- | --- | --- | --- | --- | --- | --- | --- | --- | --- | --- | --- | --- | --- | --- | --- | --- | --- | --- | --- | --- | --- | --- | --- | --- | --- | --- | --- | --- | --- | --- | --- | --- | --- | --- | --- | --- | --- | --- | --- | --- | --- |
